# Supplementary material for: Understanding rate effects in injection-induced earthquakes
Source: Nat Commun. 2020 Jun 16;11:3053. doi: 10.1038/s41467-020-16860-y (PMC7298001; doi:10.1038/s41467-020-16860-y)
Supplement: Supplementary file 1 — Supplementary Information [file 41467_2020_16860_MOESM1_ESM.pdf]

Supplementary Information for

Understanding rate effects in injection-induced earthquakes

Maryam Alghannam[1] and Ruben Juanes[1,2*]

[1]Department of Civil and Environmental Engineering,
Massachusetts Institute of Technology, Cambridge, MA 02139, USA
[2]Department of Earth, Atmospheric and Planetary Sciences,
Massachusetts Institute of Technology, Cambridge, MA 02139, USA

*To whom correspondence should be addressed; E-mail: juanes@mit.edu.

Supplementary Note 1

Derivation of the poroelastic spring–slider equations

Frictional evolution

Frictional evolution is modeled by the rate-and-state constitutive laws, which are capable of reproducing a wide range of observed seismic and aseismic fault behaviors ranging from preseismic slip and earthquake nucleation to coseismic rupture and earthquake after slip [1, 2, 3, 4]. These laws propose that frictional shear stress τ can be described as

$$\tau = \mu(V, \Theta)\Sigma', \tag{1}$$

where Σ' is the effective normal stress (the difference between total normal stress and pore pressure), μ is the coefficient of friction, V is the slider's velocity or slip rate, and Θ is a state variable with the physical interpretation of the fractional contact area that is associated with time dependent creep [5]. It is also related to the age of asperity contacts.

We adopt Ruina's [3] slip law for the coefficient of friction because it fits experimental data at variable normal stress better than Dieterich's [1] aging law [6],

$$\mu(V, \Theta) = \mu_* + \hat{a} \ln \frac{V}{V_*} + \Theta, \tag{2}$$

where $\hat{a}$ is an experimentally derived parameter, V_* is a normalizing velocity, and μ_* is a constant appropriate for steady-state at velocity V_*. Laboratory experiments on dry rocks show that a step change in normal stress results in a sudden change in the coefficient of friction followed by a displacement-dependent decay back toward the initial steady-state value [7, 5, 8, 9]. Linker and Dieterich [5] interpret this as due to a normal stress effect on the state variable and propose to model the magnitude of the sudden change as $\hat{\alpha}\dot{\Sigma}/\Sigma$. Although the model is based on step test experiments, it captures, at least qualitatively, the pressurization-weakening effect on the coefficient of friction observed from ramping

experiments by Olsson [7]. He performed laboratory tests in which the normal stress was increased at constant rate while the load point speed was held constant. He found that shear stress is a function of the normal stress rate. When the normal stress rate was increased by 10 during steady sliding, the rate of increase of shear stress with normal stress (coefficient of friction) decreased by a factor of two—a significant effect.

Here, we combine the proposed state evolution model with the effective stress principle to get

$$\dot{\Theta} = -\frac{V}{d_{\mathrm{c}}}(\Theta + \hat{b}\ln\frac{V}{V_*}) - \hat{\alpha}\frac{\dot{\Sigma}'}{\Sigma'}, \tag{3}$$

where d_{c} is the characteristic sliding distance required to replace the old contact population with a new one, $\hat{b}$ is a constitutive parameter, and $\hat{\alpha}$ is a scaling factor. Theoretical and laboratory studies for a sudden change in normal stress show that $\hat{\alpha}$ ranges from 0 to μ [5, 10, 11], but more studies are needed to determine the value of $\hat{\alpha}$ for a gradual change in normal stress. From momentum balance of forces acting on the slider, the equations of motion of the system evolution at variable effective normal stress become

$$\dot{U} = V_0 - V, \tag{4}$$

$$\dot{V} = \frac{1}{(T/2\pi)^2}\left[U - \frac{1}{k_{\mathrm{s}}}\mu(V,\Theta)\Sigma'\right], \tag{5}$$

where $T = 2\pi\sqrt{m/k_{\mathrm{s}}}$ is the vibration period of the analogous freely slipping system [12].

Poroelastic coupling

To obtain a physical evolution of effective stress on the frictional surface, we couple it with a poroelastic model of pore pressure and rock deformation. Starting with the principle of mass conservation, we specify the change of mass from fluid diffusion to be Δm_{diff}, mass accumulation due to rock expansion or fluid compressibility $\frac{\partial}{\partial t}(\rho V_{\mathrm{f}})\Delta t$, and injection source term to be $\tilde{Q}\Delta t$. We assume that both the fluid and rock matrix are

compressible [13], and so mass balance leads to

$$\Delta m_{\mathrm{diff}} = \frac{\partial(\rho V_{\mathrm{f}})}{\partial t}\Delta t - \tilde{Q}\Delta t, \tag{6}$$

where the change in fluid mass due to pressure diffusion can be written using Darcy's law as

$$\Delta m_{\mathrm{diff}} = -\frac{\rho k A(P - P_0)}{\eta L}\Delta t, \tag{7}$$

where η is fluid dynamic viscosity, k is permeability, and L is the pressure diffusion length. The mass accumulation term can be expressed as

$$\frac{\partial}{\partial t}\rho V_{\mathrm{f}}\Delta t = \frac{\partial}{\partial t}\rho(P)HA\Delta t = \frac{\partial}{\partial t}(\rho_0(1 + c_{\mathrm{f}}(P - P_0))(H_0 + W)A\Delta t, \tag{8}$$

where H is the current height of the slider, ρ_0 is the initial fluid density, c_{f} is fluid compressiblity, H_0 is the initial height of the slider, and W is the position of the piston. When fluid is injected into a rock that is free to deform in the direction orthogonal to sliding, the addition of mass induces an increase of volume equivalent to

$$V_{\mathrm{f}} - V_{\mathrm{f},0} = AW, \tag{9}$$

where $V_{\mathrm{f},0}$ is the initial fluid volume. We then derive an expression for rock deformation W from force balance, while using the convention of compression positive,

$$W = \frac{A}{k_{\mathrm{n}}}(\Sigma_0' - \Sigma + P), \tag{10}$$

where k_{n} is the normal spring stiffness and Σ_0' is the initial effective stress. We further approximate the mass accumulation term to

$$\rho\frac{\partial}{\partial t}[AH]\Delta t + AH\frac{\partial P}{\partial t}\Delta t = \rho\frac{A}{k_{\mathrm{n}}}\frac{\partial P}{\partial t}\Delta t + H\frac{\rho_0}{\rho}c_{\mathrm{f}}\frac{\partial P}{\partial t}\Delta t \approx \rho\frac{A}{k_{\mathrm{n}}}\frac{\partial P}{\partial t}\Delta t + H_0 c_{\mathrm{f}}\frac{\partial P}{\partial t}\Delta t. \tag{11}$$

Note that we consider that the total stress Σ is analogous to overburden stress in the earth, and is therefore constant in time. By substituting Eqs. (7)-(10) into Eq. (6), we

find that pore pressure satisfies a diffusion equation that leads to transient behavior at early times and steady-state behavior at late times

$$\dot{P} = \frac{k_{\mathrm{n}}^{\mathrm{eff}} k}{\eta L A}(P_0 - P) + \frac{k_{\mathrm{n}}^{\mathrm{eff}}}{A} Q, \tag{12}$$

where η is fluid dynamic viscosity ($\eta = \nu\rho$), Q is the volumetric injection rate per unit area ($Q = \tilde{Q}/\rho A$), and $k_{\mathrm{n}}^{\mathrm{eff}} = (1/k_{\mathrm{n}} + c_{\mathrm{f}} H_0/A)^{-1}$ is an effective stiffness somewhat equivalent to the uniaxial bulk modulus or the reciprocal of the uniaxial specific storage per diffusion length in a continuum [13]. Since the slider has a unit base area ($A = 1$), the evolution of the pore pressure as a result of fluid injection follows

$$\dot{P} = \frac{k_{\mathrm{n}}^{\mathrm{eff}} k}{\eta L}(P_0 - P) + k_{\mathrm{n}}^{\mathrm{eff}} Q. \tag{13}$$

Supplementary Note 2

Governing equations in dimensionless form

The equations describing the dynamic motion of the spring-poroslider system (Figure. 1B) with an evolving pore pressure, in dimensional form, are

$$\dot{U} = V_0 - V, \tag{14}$$

$$\dot{V} = \frac{1}{(T/2\pi)^2}\left[U - \frac{1}{k_{\mathrm{s}}}(\mu_* + \hat{a}\ln\frac{V}{V_*} + \Theta)(\Sigma - P)\right], \tag{15}$$

$$\dot{\Theta} = -\frac{V}{d_{\mathrm{c}}}(\Theta + \hat{b}\ln\frac{V}{V_*}) + \hat{\alpha}\frac{\dot{P}}{(\Sigma - P)}, \tag{16}$$

$$\dot{P} = \frac{k_{\mathrm{n}}^{\mathrm{eff}}k}{\eta L}(P_0 - P) + k_{\mathrm{n}}^{\mathrm{eff}}Q. \tag{17}$$

Choosing the following characteristic quantities: $u_{\mathrm{c}} = d_{\mathrm{c}}$, $v_{\mathrm{c}} = V_*$, $\mu_{\mathrm{c}} = \mu_*$, $p_{\mathrm{c}} = P_0$, $\tau_{\mathrm{c}} = \mu_*(\Sigma - P_0)$, $\theta_{\mathrm{c}} = \mu_*$, and $t_{\mathrm{c}} = d_{\mathrm{c}}/V_*$, the equations describing the dynamic motion of the system, in dimensionless form, become

$$\dot{u} = v_0 - v, \tag{18}$$

$$\dot{v} = \frac{1}{\epsilon^2}\left[u - \frac{1}{\kappa}(1 + a\ln v + \theta)(\sigma - p)\right], \tag{19}$$

$$\dot{\theta} = -v(\theta + b\ln v) + \alpha\frac{\dot{p}}{(\sigma - p)}, \tag{20}$$

$$\dot{p} = c(p_0 - p) + rq, \tag{21}$$

where $\kappa = (k_{\mathrm{s}}d_{\mathrm{c}})/\tau_{\mathrm{c}}$, $a = \hat{a}/\mu_{\mathrm{c}}$, $b = \hat{b}/\mu_{\mathrm{c}}$, $\alpha = \hat{\alpha}/\mu_{\mathrm{c}}$, $\epsilon = (T/2\pi)/t_{\mathrm{c}}$, $c = t_{\mathrm{c}}/(\eta L/k_{\mathrm{n}}^{\mathrm{eff}}/k)$, $r = t_{\mathrm{c}}k_{\mathrm{n}}^{\mathrm{eff}}$, and $q = Q/p_{\mathrm{c}}$. The parameter κ is the normalized shear stiffness, and a, b, α are normalized frictional parameters. The parameter ϵ is the normalized oscillation period or ratio of inertial to state-evolution timescales, which may range from 10^{-8} to 10^{-6} depending on rupture diameter and shear wave speed. The parameter c is the normalized diffusivity or ratio of the pore-pressure to the state-evolution timescales, which may range

from 10^{-4} to 10^{1} depending on reservoir permeability, bulk modulus (storativity), and well-fault distance. The parameter rq is the normalized injection rate, which may range from 10^{-5} to 10^{-1} depending on injection rate and reservoir size.

Supplementary Note 3

Linear stability analysis

The stability of steady frictional sliding to small perturbations in velocity depends on the state of pore pressure. For a constant pore pressure, linear stability analysis leads to the steady-state stability condition by Ruina (1983) [3], which shows that the slider is stable when the dimensionless spring stiffness of the loading system exceeds a critical value given by

$$\kappa_{\text{crit}} = (b - a)(\sigma - p), \tag{22}$$

and it is unstable otherwise. Pore pressure, however, is not constant in time, and its evolution depends on the injection rate and on the poroelastic and hydraulic parameters of the rupture (Eq. (21)). A common approach to stability analysis with time-varying state variables is the use of the quasi-steady-state approximation [14, 15, 16, 17]. Using this approach, we freeze time in the pore pressure solution and then perform a linear stability analysis of the spring–poroslider system at a fixed pore pressure.

The equations describing the quasi-static motion of the spring–poroslider system evolving at variable pore pressure p, in dimensionless form, are

$$\dot{u} = v_0 - v, \tag{23}$$

$$0 = (\sigma - p)\mu(v, \theta) - \kappa u, \tag{24}$$

$$\dot{\theta} = -v(b \ln v + \theta) + \alpha \frac{\dot{p}}{(\sigma - p)}. \tag{25}$$

The quasi-steady-state values of the variables are $v_{\text{qss}} = v_0$ and $\theta_{\text{qss}} = \alpha \dot{p}/(\sigma - p)/v_0 - b \ln v_0$. Linearizing Eqs. (23)–(25) about the quasi-steady-state, and then taking the time

derivative of Eq. (24), yields

$$\Delta\dot{v} = \frac{v_0}{a}\left[b + \frac{\alpha}{v_0}\frac{\dot{p}}{(\sigma - p)} - \frac{\kappa}{(\sigma - p)}\right]\Delta v + \frac{v_0^2}{a}\Delta\theta, \tag{26}$$

$$\Delta\dot{\theta} = -\left[b + \frac{\alpha}{v_0}\frac{\dot{p}}{(\sigma - p)}\right]\Delta v - v_0\Delta\theta. \tag{27}$$

Equations (26)–(27) represent a 2×2 system of autonomous linear ODEs with solutions of the form $\Delta v = Ve^{\lambda t}$, $\Delta\theta = \Theta e^{\lambda t}$, where λ is the growth rate and t is time. Substituting these forms into the linearized equations (26)–(27) yields the characteristic equation

$$a(\sigma - p)\lambda^2 + (-\alpha\dot{p} + \kappa v_0 - (b-a)(\sigma - p)v_0)\lambda + \kappa v_0^2 = 0. \tag{28}$$

If the real part of the roots λ_{i} are negative for all i, perturbations from the quasi-steady-state are damped and the system is stable. If the real part of the roots λ_{i} are positive for some i, then perturbations grow exponentially and the system is unstable. At $\Re(\lambda_{\mathrm{i}}) = 0$, we find that the dimensionless critical stiffness is

$$\kappa_{\mathrm{crit}} = (b-a)(\sigma - p) + \frac{\alpha}{v_0}\dot{p}. \tag{29}$$

If we express the instability condition (Eq. (29)) in terms of a critical dimensionless injection rate, above which an earthquake is induced, we obtain

$$rq_{\mathrm{crit}} = \frac{\kappa - (b-a)(\sigma - p_0)}{\frac{\alpha}{v_0}e^{-ct} - \frac{b-a}{c}(1 - e^{-ct})}. \tag{30}$$

Supplementary Note 4

Nonlinear simulations

To validate our analytical instability criterion (Eq. (29)), we simulate the fully dynamic equations of motion of the spring–poroslider system (Eqs. (18)-(21)) with the following initial conditions

$$u(t=0) = v_0(\sigma - p_0)(1 + (a-b)\ln v_0)/\kappa, \tag{31}$$

$$v(t=0) = v_0, \tag{32}$$

$$\theta(t=0) = 0, \tag{33}$$

$$p(t=0) = p_0. \tag{34}$$

These coupled nonlinear ordinary differential equations are solved in MATLAB using the `ode15s` solver for stiff systems. Representative results of motion without fluid injection and motion while fluid is being injected into the poroslider at a constant rate are shown in Supplementary Figures 1 and 2, respectively.

The simulation without fluid injection is performed using parameter values $a = 0.01$, $b = 0.02$, $\alpha = 1$, $\epsilon = 10^{-6}$, $c = 3\times10^{-2}$, and $\kappa = 0.011$. The simulation with fluid injection is performed using the same parameter values along with $rq = 5 \times 10^{-3}$. The poroslider is initially sliding steadily at a fixed loading velocity $v_0 = 1$ and a zero fluid injection rate $q = 0$. It is then made unstable by suddenly increasing the loading velocity to a new fixed value $v_0 = 1.5$ and injecting fluid at a constant rate $q = 10^{-9}$. This triggers two stick-slip events, consisting of a slow build-up followed by a sudden discharge of accumulated stress. The buildup occurs at a nearly stationary state $v \approx 0.1$ over a time period of 15, whereas the discharge is significantly faster reaching a peak velocity $v \approx 10^7$, and has a duration of 0.5 (Supplementary Figure 2A). Following the two stick-slip events, the poroslider evolves toward steady sliding at a relatively low and constant velocity $v \approx 1.5$.

The normalized stress variations with velocity, slip, and time are shown in Supplementary Figure 2B. Initially, the sudden change in load point velocity and effective normal stress creates an excess of spring force over the frictional resistance, which in turn accelerates the poroslider. The stress drops to a steady state value corresponding to slip velocity. The poroslider continues to slip at a high rate until further shortening of the spring becomes too difficult. It then decelerates to reach a nearly stationary state. During this stage, the stress builds up to a peak value corresponding to stick velocity, and the stick-slip cycle repeats. Note that the cycle is not repeated exactly, the stress buildup after a slip event is dependent on the current effective normal stress. Eventually, the decrease in effective normal stress terminates the stick-slip cycle and initiates an indefinite period of steady-sliding. The stress exhibits decaying oscillations that decrease in amplitude with time.

Supplementary Note 5

Analytical vs numerical estimates of critical stiffness

As a whole, Supplementary Figure 2 shows that the analytical critical stiffness (Eq. (29)) is in good qualitative agreement with the numerical simulation results. To validate our instability criterion quantitatively, we compare our instability criterion against estimates obtained empirically from the fully dynamic nonlinear simulations (Supplementary Figure 3). The analytical estimate (blue) works well when the growth rate of perturbations is large compared to the growth rate of the pore pressure. Initially, when pore pressure grows rapidly, estimates differ slightly, but they become indistinguishable at late times, when pore pressure changes relatively slowly. We suspect that the small difference in estimates at early times is due to, at least partially, the use of the QSSA in our analysis, which we discuss in detail in Supplementary Note 6.

Supplementary Note 6

Quasi-steady-state approximation

The quasi-steady-state approximation, in general, is an approach to simplify dynamic systems of ordinary differential equations with an initial fast transient, after which some of the dependent variables can be assumed to be in steady-state with regard to the other slowly evolving dependent variables [16]. In particular, the QSSA is a good approach to use in our analysis because it allows us to study the stability of steady frictional sliding to small perturbations in velocity while pore pressure is evolving. The sliding velocity and the velocity-dependent part of the state variable are in steady state with respect to the pore pressure. Here, we analyze the QSSA in the context of singular perturbation theory following the analysis by Segel and Slemrod (1989) [16], identify the small parameter(s) necessary for the validity of the QSSA, and quantify the error associated with it.

Reduced dimensional equations

As shown in Supplementary Note 1, the dynamics of our poroelastic spring-slider model is governed by a system of four coupled nonlinear ODEs. Under quasi-static loading, velocity is the fastest evolving variable of the system and it responds instantaneously (negligible inertia) to small perturbations. Thus we can focus our analysis on a reduced system of ODEs at steady-state velocity $V = V_0$,

$$\dot{\Theta} = -\frac{V_0}{d_c}(\Theta + \hat{b}\ln\frac{V_0}{V_*}) + \hat{\alpha}\frac{\dot{P}}{\Sigma - P}, \tag{35}$$

$$\dot{P} = \frac{k_n^{\text{eff}} k}{\eta L}(P_0 - P) + k_n^{\text{eff}} Q, \tag{36}$$

with initial conditions

$$\Theta(0) = 0, \tag{37}$$

$$P(0) = P_0. \tag{38}$$

Timescales

As a first step in the analysis, we estimate the fast timescale t_Θ of the pre-steady-state period and the slow timescale t_P for the evolution of pore pressure. To estimate t_Θ we make the approximation $P \approx P_0$ in Eq. (35). The solution for the state variable becomes

$$\Theta(t) = \bar{\Theta}(e^{-\frac{V_0}{d_\mathrm{c}}t} - 1), \tag{39}$$

where $\bar{\Theta} = \hat{b} \ln V_0/V_* - \hat{\alpha}(d_\mathrm{c}/V_0)(\dot{P}_0/(\Sigma - P_0))$. Subsequently, we take

$$t_\Theta = \frac{d_\mathrm{c}}{V_0}. \tag{40}$$

Since the pore pressure evolution is independent of the evolution of the state variable Θ, we estimate t_P by solving Eq. (36) with the initial condition $P(0) = P_0$ to obtain

$$P(t) = P_0 + \frac{\eta L}{k} Q(1 - e^{-\frac{k_\mathrm{n}^\mathrm{eff} k}{\eta L}t}). \tag{41}$$

Similarly, we take

$$t_\mathrm{P} = \frac{\eta L}{k_\mathrm{n}^\mathrm{eff} k}. \tag{42}$$

Scaled dimensionless equations

During the pre-steady-state, it is reasonable to scale time by t_Θ, where the dimensionless time τ is given by

$$\tau = \frac{t}{t_\Theta}. \tag{43}$$

Thus, the scaled dimensionless governing equations become

$$\frac{\partial \theta}{\partial \tau} = -v_0(\theta + b \ln v_0) + \frac{\alpha}{\sigma - p}\frac{\partial p}{\partial \tau}, \tag{44}$$

$$\frac{\partial p}{\partial \tau} = \frac{t_\Theta}{t_\mathrm{P}}(p_0 - p) + t_\Theta q, \tag{45}$$

with initial conditions

$$\theta(0) = 0, \tag{46}$$

$$p(0) = p_0, \tag{47}$$

where we have defined $b = \hat{b}/\mu_0$, $\alpha = \hat{\alpha}/\mu_0$, and $q = Q/p_0$. After the pre-steady state, the QSSA is assumed to be valid and t_P becomes a reasonable timescale. We introduce a new dimensionless scaled time $\tilde{t}$ by

$$\tilde{t} = \frac{t}{t_\mathrm{P}}, \tag{48}$$

with which the scaled dimensionless governing equations become

$$\frac{t_\Theta}{t_\mathrm{P}}\frac{\partial \theta}{\partial \tilde{t}} = -v_0(\theta + b \ln v_0) + \frac{t_\Theta}{t_\mathrm{P}}\frac{\alpha}{\sigma - p}\frac{\partial p}{\partial \tilde{t}}, \tag{49}$$

$$\frac{\partial p}{\partial \tilde{t}} = (p_0 - p) + t_\mathrm{P} q. \tag{50}$$

Singular perturbation

Approximate solutions can now be obtained by methods of singular perturbation theory [18], for $0 < t_\Theta/t_\mathrm{P} \ll 1$. A solution of Eqs. (44)-(45) is obtained of the form

$$\theta(\tau) = \theta^{(0)}(\tau) + \frac{t_\Theta}{t_\mathrm{P}}\, \theta^{(1)}(\tau) + ..., \tag{51}$$

$$p(\tau) = p^{(0)}(\tau) + \frac{t_\Theta}{t_\mathrm{P}}\, p^{(1)}(\tau) + ..., \tag{52}$$

where

$$\theta^{(0)}(\tau) = \bar{\theta}(e^{-\tau} - 1), \tag{53}$$

$$p^{(0)}(\tau) = p_0. \tag{54}$$

Similarly, the solution of Eqs. (49)-(50) obtained of the form

$$\theta(\tilde{t}) = \theta_0(\tilde{t}) + \frac{t_\Theta}{t_\mathrm{P}}\,\theta_1(\tilde{t}) + \dots\,, \tag{55}$$

$$p(\tilde{t}) = p_0(\tilde{t}) + \frac{t_\Theta}{t_\mathrm{P}} p_1(\tilde{t}) + \dots\,, \tag{56}$$

where

$$\theta_0 = -b \ln v_0 + \frac{\alpha}{v_0}\frac{t_\Theta q}{\sigma - p_0}, \tag{57}$$

$$\frac{\partial p_0}{\partial \tilde{t}} = t_\mathrm{P} q. \tag{58}$$

Note that Eqs. (57)-(58) are associated with initial conditions Eqs. (37)-(38), and represent the initial state about which we linearized the spring-poroslider system (section 3). The results of this analysis remain valid for general initial conditions $\Theta(0) = \Theta_\mathrm{i}$ and $P(0) = P_\mathrm{i}$ [16], where Θ_i ranges from 0 to Θ_ss and P_i ranges from P_0 to P_ss. Linearizing the spring-poroslider system about the true steady-state, where

$$\theta_0 = -b \ln v_0, \tag{59}$$

$$\frac{\partial p_0}{\partial \tilde{t}} = 0, \tag{60}$$

thus yields the Ruina (1983) stability condition [3].

QSSA validity conditions

A necessary aspect of the QSSA is that the duration of the pre-steady-state period is much shorter than the characterstic time for the pore pressure evolution. An essential condition for the QSSA to be valid after the pre-steady state is therefore $t_\Theta \ll t_\mathrm{P}$,

$$0 < c = \left(\frac{d_\mathrm{c}}{V_0}\right) \Big/ \left(\frac{\eta L}{k_\mathrm{n}^\mathrm{eff} k}\right) \ll 1. \tag{61}$$

Note that the initial condition $P(0) = P_0$ is reasonable for the QSSA only if there is a negligible relative change $|\Delta P/P_0|$ in pore pressure during the pre-steady state. We

estimate $|\Delta P/P_0|$ by

$$\left|\frac{\Delta P}{P_0}\right| \approx \frac{1}{P_0}\left|\frac{\partial P}{\partial t}\right|_{\max} t_\Theta. \tag{62}$$

An additional condition for the validity of the QSSA is therefore

$$0 < rq = \frac{d_\mathrm{c} k_\mathrm{n}^\mathrm{eff}}{V_0}\frac{Q}{P_0} \ll 1. \tag{63}$$

Recall that parameter c is the normalized diffusivity or ratio of the pore pressure to the state evolution timescales and parameter rq is the normalized injection rate, where $r = t_\Theta k_\mathrm{n}^\mathrm{eff}$ and $q = Q/P_0$.

Error estimates

This analysis shows that using the QSSA to study the stability of steady frictional sliding to small perturbations in velocity with an evolving pore pressure is justified when conditions Eqs. (61) and (63) are met. In other words, if in a timescale t_Θ sliding reaches steady state with a constant pore pressure, then assuming that sliding is in a quasi-steady state with a changing pore pressure is valid when the pore pressure change occurs on a time scale t_P that is long compared to t_Θ and $\Delta P|_{t_\Theta}$ is small compared to P_0.

Therefore, we expect that the accuracy of our instability criterion depends on dimensionless parameters c and rq. Here we evaluate the error in the analytical estimate of the critical stiffness required to trigger the first slip event (Supplementary Figure 4). We indeed find that the error decreases as c or rq decrease. It becomes small ($< 15\%$) when the normalized diffusivity and normalized injection rate reach small values ($c \leq 5 \times 10^{-2},\ rq \leq 5 \times 10^{-3}$). It is also interesting to note that the QSSA validity may be extended to instances where c is of order one provided that rq is significantly smaller than one (green curve).

Supplementary Note 7

Application to the Denver earthquakes

To bridge the gap between the analysis of the idealized spring-poroslider model and the real world, we express our instability criterion in dimensional form, and identify values of dimensionless parameters c and rq that correspond to real-world settings.

In dimensional form, the critical stiffness from the linear stability analysis is

$$k_{\mathrm{s,crit}} = \frac{(\hat{b} - \hat{a})}{d_{\mathrm{c}}}(\Sigma - P) + \frac{\hat{\alpha}}{V_0}\dot{P}, \tag{64}$$

or, equivalently,

$$k_{\mathrm{s,crit}} = \left[(\hat{b} - \hat{a}) + \hat{\alpha}\frac{d_{\mathrm{c}}}{V_0}\frac{\dot{P}}{(\Sigma - P)}\right]\frac{(\Sigma - P)}{d_{\mathrm{c}}}, \tag{65}$$

where the term $\hat{b} - \hat{a}$ represents the original velocity weakening effect and the dimensionless term $\hat{\alpha}(d_{\mathrm{c}}/V_0)\dot{P}/(\Sigma - P)$ represents an additional weakening effect from fluid pressurization. Note that this pressurization term is maximum at early times and is approximately equal to rq.

The 1960s Denver earthquakes is a good example of a real-world setting, where it is well-documented that injection of wastewater into the fractured Precambrian granite gneiss underneath the Rocky Mountain Arsenal triggered the earthquakes and where injection rate is directly related to the frequency of earthquakes [19, 20]. The reservoir spans a depth interval from 3.7 to 7 km below the surface. Experimental data on granite at this depth shows velocity weakening behavior ($\hat{b} - \hat{a}$ in the range 0.002 to 0.005, $\mu_0 = 0.7$ to 0.75) [21].

To identify values of dimensionless parameter c that correspond to this setting, we estimate the state evolution timescale t_Θ and the pore pressure evolution timescale t_{p}:

$$c = \frac{t_\Theta}{t_{\mathrm{P}}}. \tag{66}$$

The state evolution timescale t_Θ is

$$t_\Theta = \frac{d_c}{V_0}. \tag{67}$$

We find that t_Θ ranges from 10 days to 4 months based on field data of the characteristic slip distance and loading rate ($d_\mathrm{c} = 10^{-3}$ to 10^{-2} m, $V_0 = 10^{-9}$ m s^{-1}) [22, 4].

The pore pressure evolution time scale t_P can be transferred from the poroslider model to field settings,

$$t_\mathrm{P} = \underbrace{\frac{\eta L}{k_\mathrm{n}^\mathrm{eff} k}}_{\text{poroslider}} = \frac{L}{k/\eta}\left[\frac{1}{k_\mathrm{n}} + H_0 c_\mathrm{f}\right] = \frac{L^2}{k/\eta}\left[\frac{1}{K_\mathrm{v}} + \phi\frac{1}{K_\mathrm{f}}\right] = \frac{L^2}{k/\eta}\frac{S_\mathrm{s}}{\rho g} = \underbrace{\frac{L^2 S}{T}}_{\text{field}}. \tag{68}$$

We find that t_P is approximately 2 years based on a reservoir analysis of the Denver earthquakes, with transmissivity $T = 10^{-5}$ m^2 s^{-1}, storativity $S = 10^{-5}$, and characteristic length scale $L = 8 \times 10^3$ m [23].

In a similar manner, we identify values of dimensionless parameter rq. We translate this quantity from the poroslider model to field settings:

$$rq = \underbrace{\frac{d_\mathrm{c}}{V_0} k_\mathrm{n}^\mathrm{eff} \frac{Q}{P_0}}_{\text{poroslider}} = \frac{d_\mathrm{c}}{V_0} k_\mathrm{n}^\mathrm{eff} \frac{Q_\mathrm{w}}{P_0 W B} = \frac{d_\mathrm{c}}{V_0} \frac{\left[\frac{1}{K_\mathrm{v}} + \phi\frac{1}{K_\mathrm{f}}\right]^{-1}}{L} \frac{Q_\mathrm{w}}{P_0 W B} = \underbrace{\frac{d_\mathrm{c}}{V_0}\frac{\rho g}{LS}\frac{Q_\mathrm{w}}{P_0 W}}_{\text{field}}, \tag{69}$$

and evaluate values based on the reservoir analysis and injection data, with reservoir pressure $P_0 = 30$ MPa, reservoir width $W = 3 \times 10^3$ m, and field injection rate $Q_\mathrm{w} = 2$ to 9 million gal mo^{-1} [19, 23].

Therefore, reasonable estimates of c and rq for this setting would be in the order of 10^{-2} to 10^{-1} and 10^{-3} to 10^{-1}, respectively. Note that both estimates are much smaller than one, and thus meet the QSSA validity conditions (Supplementary Figure 5).

Having determined the validity of the QSSA analysis to this setting, we now assess whether pressurization rate effects were likely significant during fluid injection leading

to the Denver earthquakes. In dimensionless form, the critical stiffness κ_{crit} is given by Eq. (S29). Prior to fluid injection, the pore pressure is constant and the critical stiffness,

$$\kappa_{\text{crit}} = (b - a)(\sigma - p_0), \tag{70}$$

is estimated to be 0.005 ($b - a = 0.003$ to 0.007, $\sigma - p_0 = 1$). Shortly following the start of fluid injection, the pore pressure increases rapidly and the dimensionless critical stiffness takes the form:

$$\kappa_{\text{crit}}\Big|_{t=0} = (b - a)(\sigma - p_0) + \frac{\alpha}{v_0}\dot{p}\Big|_{t=0} = (b - a)(\sigma - p_0) + \frac{\alpha}{v_0} rq. \tag{71}$$

This results in an increase in critical stiffness at early times of around 300% ($\alpha = 1$, $v_0 = 1$, $b - a = 0.005$, $rq = 10^{-2}$), thus indicating that the additional weakening effect from fluid pressurization is likely significant in this setting.

Supplementary Note 8

Phase diagram of injection-induced seismicity

To study the influence of reservoir properties on injection-induced seismicity, we simulate the occurrence of earthquakes as a function of dimensionless injection rate $rq = d_\mathrm{c}k_\mathrm{n}^\mathrm{eff}Q/(p_\mathrm{c}V_*)$ and normalized diffusivity $c = d_\mathrm{c}k_\mathrm{n}^\mathrm{eff}k/(\eta L V_*)$ for the case $a = 0$, $b = 0.01$, $\alpha = 1$, and $\kappa = 0.011$ (Supplementary Figure 6). Diffusivity is varied by varying permeability. Each point on the phase diagram represents the maximum slip velocity of a different simulation run with a particular injection rate and a particular normalized diffusivity. The red dots indicate the occurrence of one or more earthquakes over a finite time period, whereas the grey dots indicate the absence of earthquakes over the same period. We observe two distinct regimes depending on the normalized diffusivity: a high diffusivity regime for $c > 1$, and a low diffusivity regime for $c < 0.01$.

When the normalized diffusivity is higher than one, the dimensionless pressure diffusion time is less than one. Pore pressure reaches steady-state on a very short time scale, and so the rate of change in pore pressure is negligible. To quantify this, we write

$$\dot{p} \approx 0, \tag{72}$$

$$p \approx p_0 + \frac{rq}{c}. \tag{73}$$

It is helpful to express the condition for instability (Eq. (29)) in terms of rq_crit so that an earthquake is triggered if the injection rate is higher than a critical value given by

$$rq_\mathrm{crit} \approx \left[(\sigma - p_0) - \frac{\kappa}{(b-a)}\right]c. \tag{74}$$

We find that the injection rate required to trigger an earthquake is proportional to diffusivity c, which explains the simulation results in Supplementary Figure 6: In the regime of high diffusivity $c > 1$, $q_\mathrm{crit} \sim c$. Accordingly, earthquakes are more easily triggered

when fluid is injected into a low permeability reservoir rock than a high permeability, for a fixed effective stiffness.

Conversely, when the normalized diffusivity is lower than 0.001, the dimensionless pressure diffusion time is more than 100. Pore pressure stays near the initial transient-state throughout the simulation period and so the magnitude of change in pore pressure is negligible. To quantify this, we write

$$\dot{p} \approx rq, \tag{75}$$

$$p \approx p_0. \tag{76}$$

If we express the condition for instability in terms of rq_{crit}, similar to the high diffusivity case above, we find that the injection rate required to trigger an earthquake is

$$rq_{\text{crit}} \approx \frac{v_0}{\alpha}\left[\kappa - (b-a)(\sigma - p_0)\right]. \tag{77}$$

This also explains the simulation results in Supplementary Figure 6: In the regime of low diffusivity $c < 0.01$, $q_{\text{crit}} \sim$ const. Accordingly, in this regime, earthquake triggering is independent of permeability. These results, as a whole, suggest that reservoirs with high hydraulic diffusivity and low stiffness may be safer sites for fluid injection operations compared to sites with low hydraulic diffusivity and high stiffness.

Supplementary Note 9

Earthquake likelihood vs. injection duration

Supplementary Figure 7 shows the likelihood of earthquakes as a function of the duration of the injection period for a fixed total injected volume. As can be seen, the earthquake likelihood, inferred from the peak critical stiffness, is strongly dependent on the duration of injection. A shorter injection duration or, equivalently, a higher injection rate results in a higher likelihood of earthquake triggering.

Supplementary Figures

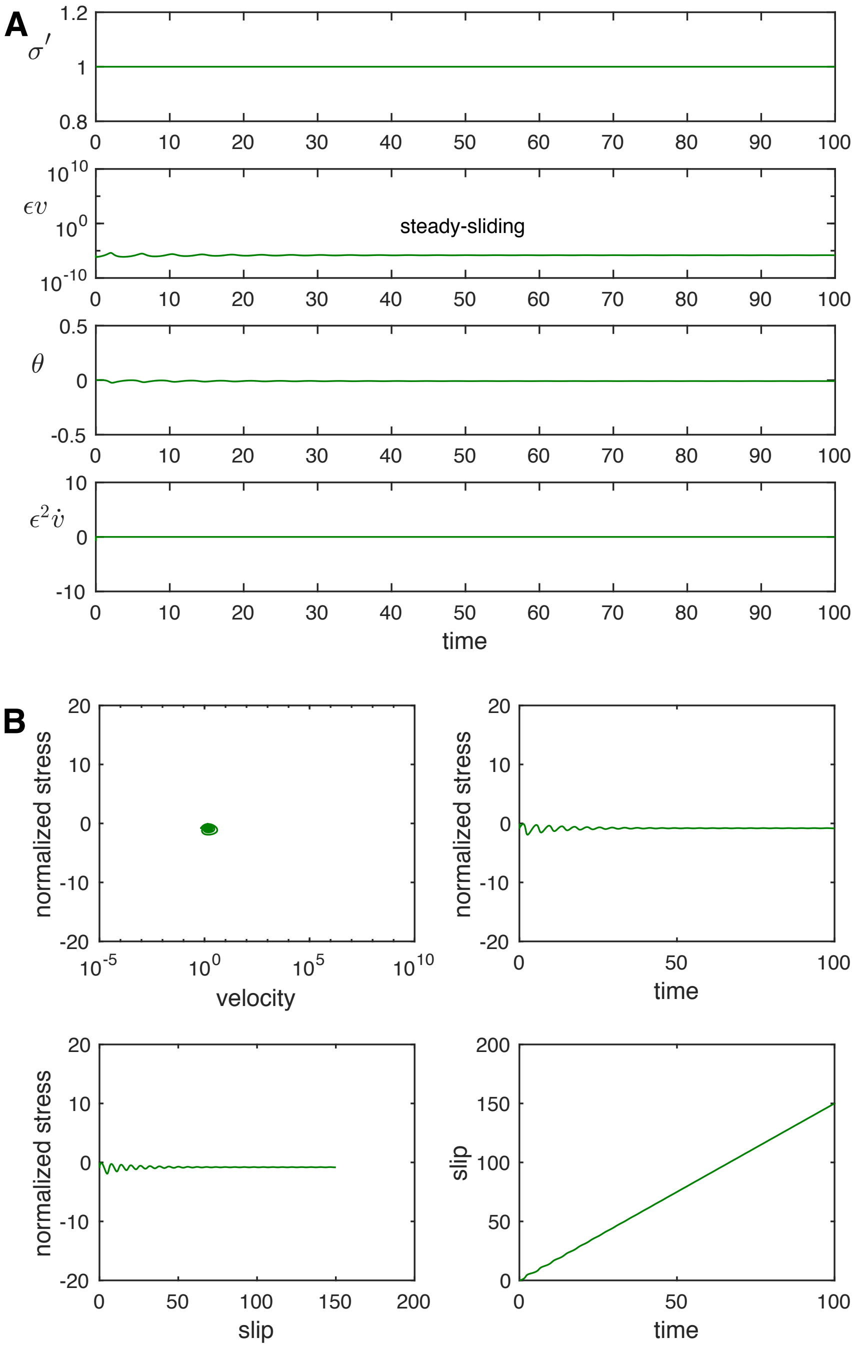

Supplementary Figure 1: Dynamics of the spring-poroslider system without fluid injection, for velocity-weakening friction. Top figure (A) shows time evolution of normal effective stress $(\sigma - p)$, velocity ϵv, state variable θ, and magnitude of the inertia term $\epsilon \dot{v}$. Bottom figure (B) shows plots of normalized stress as a function of velocity, time, and slip. Red curves represent the slip phase, blue curves represent the stick phase, and green curves represent steady-sliding.

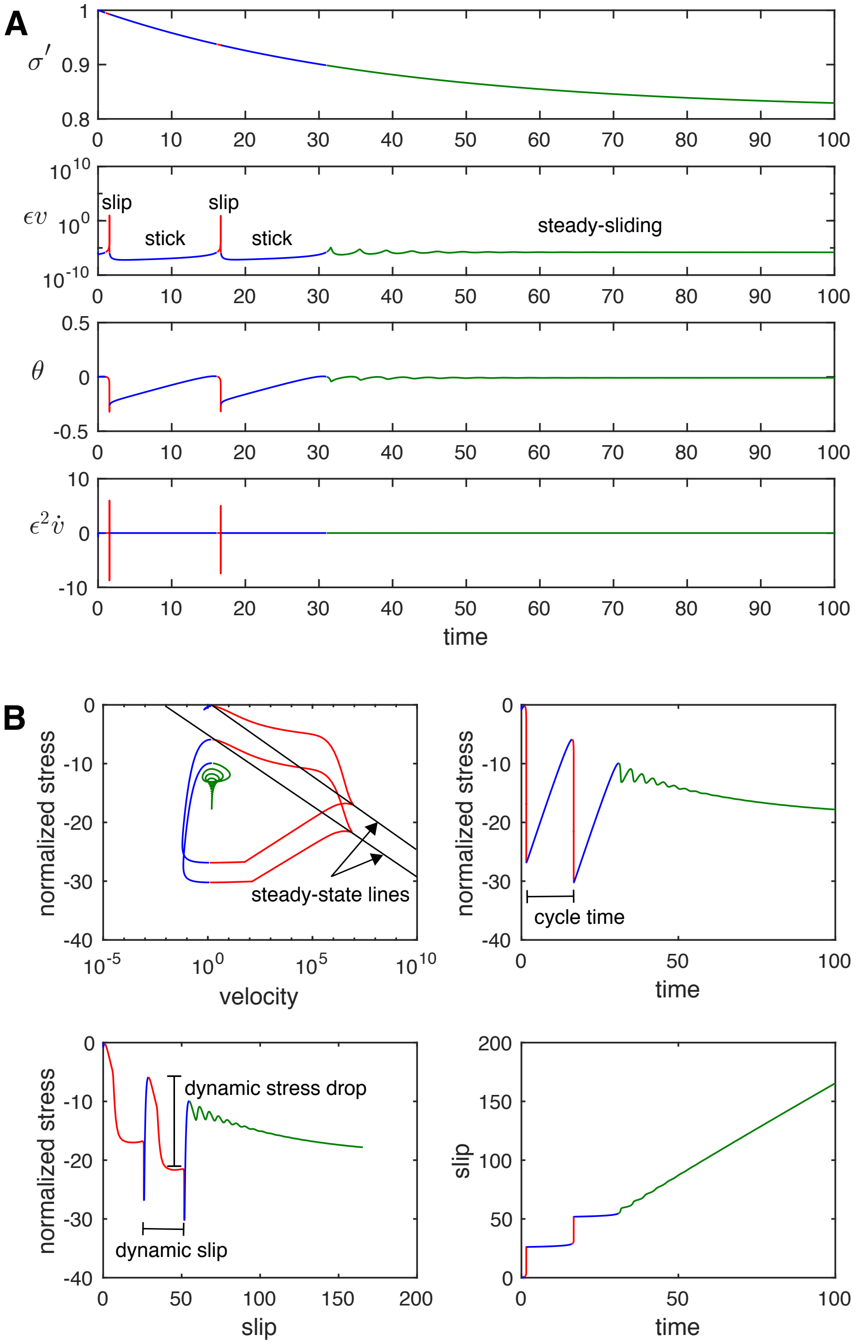

Supplementary Figure 2: Dynamics of the spring-poroslider system under constant fluid injection rate, for velocity-weakening friction. Top figure (A) shows time evolution of normal effective stress ($\sigma - p$), velocity ϵv, state variable θ, and magnitude of the inertia term $\epsilon \dot{v}$. Bottom figure (B) shows plots of normalized stress as a function of velocity, time, and slip. Red curves represent the slip phase, blue curves represent the stick phase, and green curves represent steady-sliding.

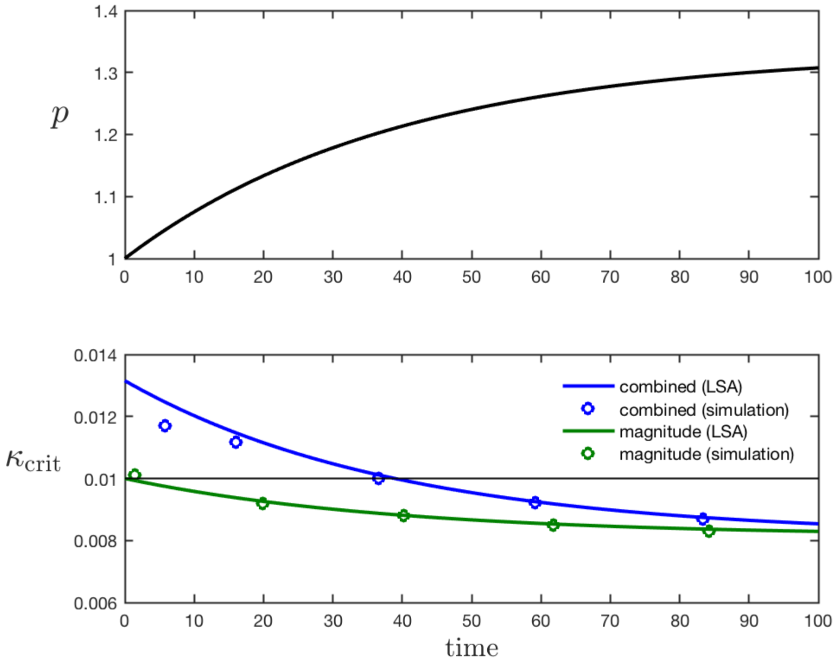

Supplementary Figure 3: Analytical vs. numerical estimates of critical stiffness $\kappa_{\rm crit}$ for a case with normalized diffusivity of $c = 3 \times 10^{-2}$ and normalized injection rate of $rq = 5 \times 10^{-3}$. Solid line represents the analytical estimate, and open circles represent numerical ones. Blue represents the combined rate and magnitude effect (setting $\alpha = 1$), and green represents the magnitude effect (setting $\alpha = 0$). The black horizontal line represents $\kappa_{\rm crit}$ prior to fluid injection.

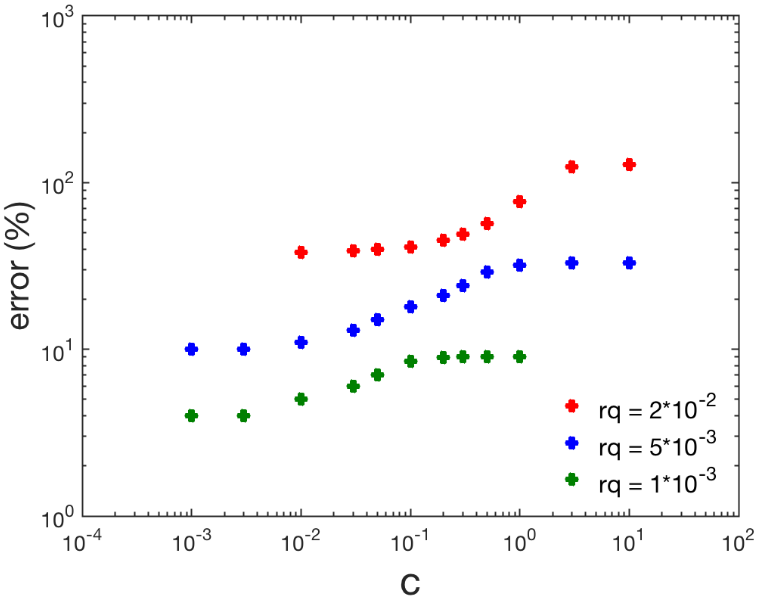

Supplementary Figure 4: Error in the instability criterion as a function of c and rq. The error is taken as the ratio of the maximum difference between the analytical and numerical estimates of the critical stiffness required to trigger the first slip event to the numerical estimate. The dimensionless parameter c is varied by varying permeability k, and rq is varied by varying injection rate Q.

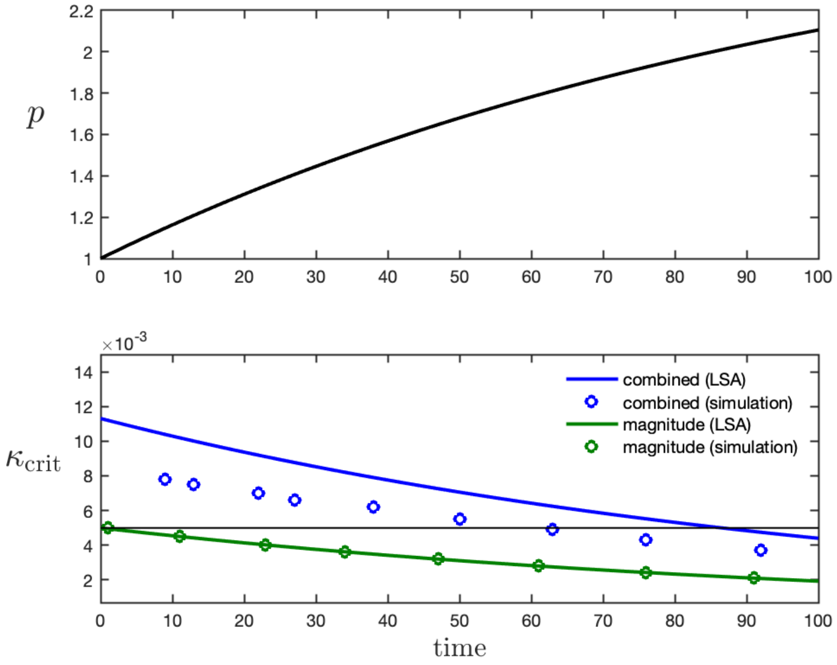

Supplementary Figure 5: Analytical vs. numerical estimates of critical stiffness κ_{crit} for a case with normalized diffusivity of $c = 1 \times 10^{-2}$ and normalized injection rate of $rq = 1 \times 10^{-2}$. Solid line represents the analytical estimate, and open circles represent numerical ones. Blue represents the combined rate and magnitude effect (setting $\alpha = 1$), and green represents the magnitude effect (setting $\alpha = 0$). The black horizontal line represents κ_{crit} prior to fluid injection.

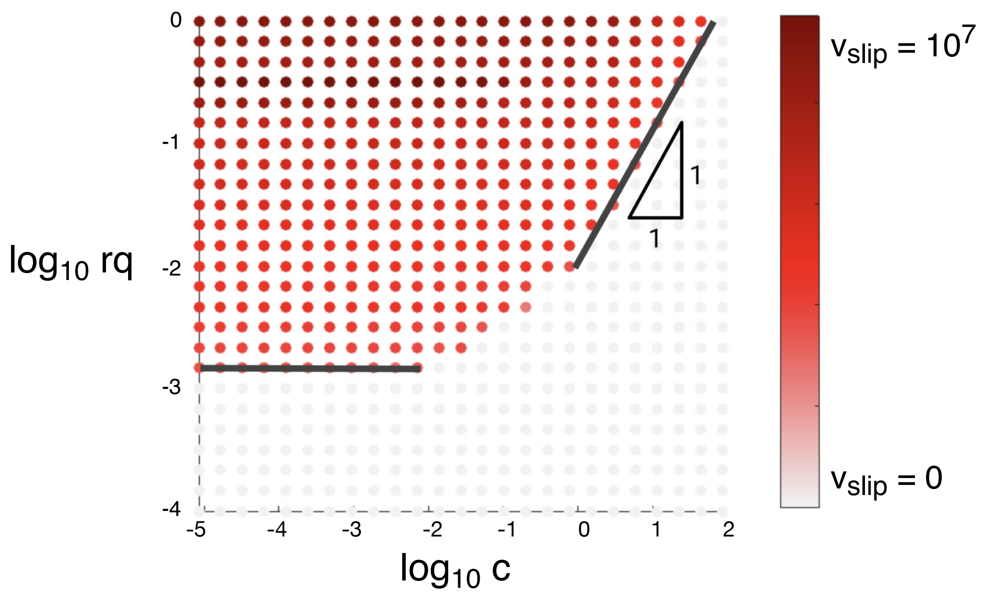

Supplementary Figure 6: Phase diagram of triggered earthquakes as a function of dimensionless injection rate rq and normalized diffusivity c. Diffusivity is varied by varying permeability k. The dots show the maximum slip rate in log scale, where red represents occurrence of one or more earthquakes during a finite simulation period, and grey represents the absence of earthquakes over the same time period.

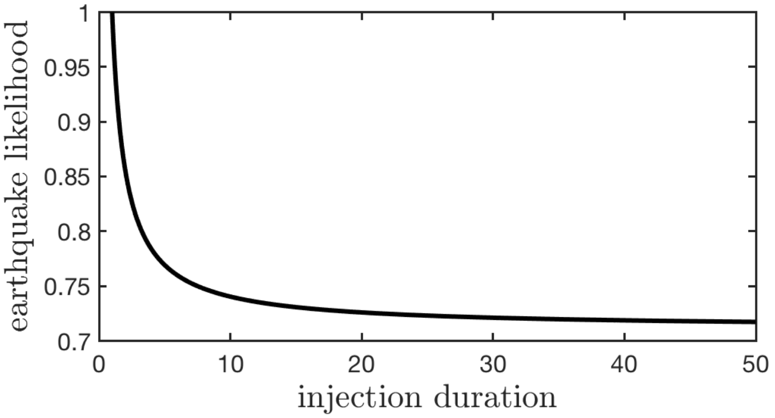

Supplementary Figure 7: Earthquake likelihood vs. injection duration. The earthquake likelihood is computed as a normalized initial critical stiffness for a given injection duration ($\kappa_{\mathrm{crit}}|_{\mathrm{T}}/\kappa_{\mathrm{crit}}|_{\mathrm{T_{min}}}$). The injection duration is computed as the ratio of a fixed total injection volume to the injection rate ($T = V/q$).

Supplementary References

1. Dieterich, J. H. Modeling of rock friction: 1. Experimental results and constitutive equations. *Journal of Geophysical Research: Solid Earth* **84**, 2161–2168 (1979).

2. Dieterich, J. H. Modeling of rock friction: 2. Simulation of preseismic slip. *Journal of Geophysical Research: Solid Earth* **84**, 2169–2175 (1979).

3. Ruina, A. Slip instability and state variable friction laws. *Journal of Geophysical Research: Solid Earth* **88**, 10359–10370 (1983).

4. Marone, C. Laboratory-derived friction laws and their application to seismic faulting. *Annual Review of Earth and Planetary Sciences* **26**, 643–696 (1998).

5. Linker, M. & Dieterich, J. H. Effects of variable normal stress on rock friction: Observations and constitutive equations. *Journal of Geophysical Research: Solid Earth* **97**, 4923–4940 (1992).

6. Richardson, E. & Marone, C. Effects of normal stress vibrations on frictional healing. *Journal of Geophysical Research: Solid Earth* **104**, 28859–28878 (1999).

7. Olsson, W. A. The effects of normal stress history on rock friction. In *The 29th US Symposium on Rock Mechanics*, 111–117 (American Rock Mechanics Association, 1988).

8. Wang, W. & Scholz, C. Micromechanics of the velocity and normal stress dependence of rock friction. *Pure and Applied Geophysics* **143**, 303–315 (1994).

9. Kilgore, B., Lozos, J., Beeler, N. & Oglesby, D. Laboratory observations of fault strength in response to changes in normal stress. *Journal of Applied Mechanics* **79**, 031007 (2012).

10. Perfettini, H., Schmittbuhl, J., Rice, J. R. & Cocco, M. Frictional response induced by time-dependent fluctuations of the normal loading. *Journal of Geophysical Research: Solid Earth* **106**, 13455–13472 (2001).

11. Hong, T. & Marone, C. Effects of normal stress perturbations on the frictional properties of simulated faults. *Geochemistry, Geophysics, Geosystems* **6**, Q03012 (2005).

12. Rice, J. R. & Tse, S. T. Dynamic motion of a single degree of freedom system following a rate and state dependent friction law. *Journal of Geophysical Research: Solid Earth* **91**, 521–530 (1986).

13. Wang, H. F. *Theory of linear poroelasticity with applications to geomechanics and hydrogeology* (Princeton University Press, 2000).

14. Lick, W. The instability of a fluid layer with time-dependent heating. *Journal of Fluid Mechanics* **21**, 565–576 (1965).

15. Robinson, J. Theoretical analysis of convective instability of a growing horizontal thermal boundary layer. *The Physics of Fluids* **19**, 778–791 (1976).

16. Segel, L. A. & Slemrod, M. The quasi-steady-state assumption: a case study in perturbation. *SIAM Review* **31**, 446–477 (1989).

17. Rao, C. V. & Arkin, A. P. Stochastic chemical kinetics and the quasi-steady-state assumption: Application to the Gillespie algorithm. *The Journal of Chemical Physics* **118**, 4999–5010 (2003).

18. Lin, C.-C. & Segel, L. A. *Mathematics applied to deterministic problems in the natural sciences* (SIAM, 1988).

19. Evans, D. M. The Denver area earthquakes and the Rocky Mountain Arsenal disposal well. *The Mountain Geologist* **3**, 23–36 (1966).

20. Healy, J., Rubey, W., Griggs, D. & Raleigh, C. The Denver earthquakes. *Science* **161**, 1301–1310 (1968).

21. Blanpied, M., Lockner, D. & Byerlee, J. Fault stability inferred from granite sliding experiments at hydrothermal conditions. *Geophysical Research Letters* **18**, 609–612 (1991).

22. Scholz, C. The critical slip distance for seismic faulting. *Nature* **336**, 761 (1988).

23. Hsieh, P. A. & Bredehoeft, J. D. A reservoir analysis of the Denver earthquakes: A case of induced seismicity. *Journal of Geophysical Research: Solid Earth* **86**, 903–920 (1981).
